# Neuropathologie I: Muskuläre Erkrankungen

**DOI:** 10.1007/s00292-022-01163-4

**Published:** 2022-12-02

**Authors:** Anne Schänzer, Carsten Dittmayer, Stefan Porubsky, Joachim Weis, Hans-Hilmar Goebel, Werner Stenzel

**Affiliations:** 1 Institut für Neuropathologie, Justus-Liebig-Universität Gießen, Arndstr. 16, 35392 Gießen, Deutschland; 2 Institut für Neuropathologie, Charité – Universitätsmedizin Berlin, corporate member of Freie Universität Berlin und Humboldt-Universität zu Berlin, 10117 Berlin, Deutschland; 3 Institut für Pathologie, JGU Universitätsmedizin Mainz, 55131 Mainz, Deutschland; 4 Institut für Neuropathologie, Universitätsklinikum der RWTH Aachen, 52074 Aachen, Deutschland; 5 Abteilung für Neuropathologie, JGU Universitätsmedizin Mainz, 55131 Mainz, Deutschland

**Keywords:** Elektronenmikroskopie, Kardiomyopathie, Myositis, Mitochondrien, Vakuolen, Electron microscopy, Cardiomyopathy, Myositis, Mitochondria, Vacuoles

## Abstract

Muskelerkrankungen umfassen hereditäre genetische und erworbene Erkrankungen, welche sowohl im Kindes- als auch im Erwachsenenalter auftreten. Bei den unterschiedlichen Muskelerkrankungen kann es ultrastrukturelle Besonderheiten geben, welche helfen, die Erkrankung weiter einzugrenzen. Spezifische Veränderungen der Sarkomerenstruktur helfen bei der Einordnung einer kongenitalen Myopathie. Die Detektion von zellulären Aggregaten unterstützt die Klassifizierung einer Myositis. Pathologisch veränderte Mitochondrien können dagegen sowohl bei genetisch bedingten Mitochondriopathien, aber auch sekundär bei erworbenen Muskelerkrankungen auftreten, wie z. B. einer Myositis. Die ultrastrukturelle Beurteilung der Herzmuskulatur kann insbesondere bei kindlichen hereditären Kardiomyopathien die Erkrankung weiter eingrenzen. Dieser Übersichtsartikel stellt die ultrastrukturellen Besonderheiten bei den unterschiedlichen Muskelerkrankungen heraus, wobei insbesondere auf pathognomonische Befunde bei bestimmten Krankheitsgruppen eingegangen wird.

## Abstract (en)

Muscle diseases include hereditary and acquired diseases with clinical manifestation in both childhood and adulthood. The different muscle diseases may have ultrastructural alterations that help us further understand the pathology of the disease. Specific changes in sarcomere structure help to classify a congenital myopathy. The detection of cellular aggregates supports the classification of myositis. Pathologically altered mitochondria, on the other hand, can occur both in genetic mitochondriopathies but also secondarily in acquired muscle diseases like myositis. Ultrastructural analysis of the myocardium is also helpful in the diagnosis of hereditary cardiomyopathies in childhood. This review article highlights the ultrastructural features of different muscle diseases and pathognomonic findings in specific disease groups.

Die pathologische Zuordnung einer Muskelerkrankung ist von großer Bedeutung, um die besten therapeutischen Möglichkeiten für Patienten bereitzustellen. Nicht immer sind die klinischen und genetischen Befunde eindeutig oder konklusiv. Daher hat die morphologische Beurteilung einer Muskelbiopsie weiterhin einen hohen Stellenwert in der Diagnostik neuromuskulärer Erkrankungen. Neben der Aufarbeitung mit speziellen enzymatischen und immunhistochemischen Techniken, ist hierbei die elektronenmikroskopische Beurteilung hilfreich, eine Diagnose zu stützen und die Pathogenese besser einzugrenzen.

Bei der Diagnostik muskulärer Erkrankungen hat die ultrastrukturelle Analyse immer noch einen hohen Stellenwert. Die Bearbeitung der Proben für Transmissionselektronenmikroskopie (TEM) ist trotz Automatisierung allerdings sehr zeitaufwendig. Weiterhin erfordert die Beurteilung eine hohe Expertise und wird daher nicht mehr in allen neuropathologischen Instituten durchgeführt. In diesem Übersichtsartikel wollen wir die ultrastrukturellen Besonderheiten bei den unterschiedlichen Muskelerkrankungen des Kindes- und Erwachsenenalters herausstellen, welche helfen sollen, die Pathogenese der Erkrankung weiter einzugrenzen. Dabei gehen wir insbesondere auf pathognomonische Befunde bei bestimmten Krankheitsgruppen ein.

Die Myopathien umfassen hereditäre und erworbene Erkrankungen, welche sowohl im Kindes- als auch im Erwachsenenalter auftreten. In den letzten Jahrzehnten haben sich die therapeutischen Möglichkeiten dieser „rare diseases“ maßgeblich verbessert. Insbesondere Patienten mit genetischen Muskelerkrankungen stehen neue Therapien zur Verfügung. Daher ist eine präzise Diagnose der Erkrankung von großer Wichtigkeit. Die klinischen und genetischen Befunde sind jedoch nicht in jedem Fall richtungsweisend. Die Muskelpathologie kann in diesen Fällen hilfreich sein, die zugrunde liegende Ursache der Erkrankung aufzuarbeiten bzw. Hilfsstellung für eine molekulare Paneldiagnostik zu geben. Zusätzlich können die Befunde die Beurteilung, ob eine neue Genvariante als pathogen einzuordnen ist, erleichtern.

Obwohl es auch ultrastrukturelle Veränderungen gibt, die für spezifische Erkrankungen pathognomonisch sind, sollten die Befunde immer im Gesamtkontext aller histologischen und klinischen Befunde interpretiert werden. Neben enzymatischen und immunhistochemischen Reaktionen an unfixiertem Muskelgewebe sollte daher immer Gewebe für die ultrastrukturelle Analyse in Glutaraldehyd fixiert werden [7].

## Gewebepräparation

Wie bei allen ultrastrukturellen Analysen ist die Qualität des Gewebes entscheidend für die präzise Einordnung der morphologischen Veränderungen. Kontakt zu Kochsalzlösung sowie Quetsch- und Zugtraumata können die Beurteilung der Muskulatur stark beeinträchtigen. Eine Umbettung aus Paraffinmaterial ist möglich, aber nicht ideal. Postmortale Veränderungen setzen in der quergestreiften Muskulatur sehr rasch ein, wobei insbesondere Mitochondrien betroffen sind. Dies sollte bei einer Obduktion mit entsprechender Fragestellung beachtet werden. Von der nativen Muskelprobe wird ein längliches Fragment von ca. 0,3 × 0,1 × 0,1 cm mit longitudinaler Muskelfaserausrichtung mit der Rasierklinge vorsichtig präpariert. Dabei sollte man randständige Anteile aufgrund von Quetschartefakten nicht für die Einbettung verwenden. Auch kleine Nadelproben sind geeignet. Das Muskelgewebe wird in 2–4 % Glutaraldehyd für mindestens 1,5–2 h bei Raumtemperatur fixiert und dann in Puffer gewaschen. Das Gewebe kann bei 4 °C für mehrere Tage bis Wochen in phosphathaltiger Pufferlösung PBS aufbewahrt werden, bevor es später für die Kunstharzeinbettung verarbeitet wird. Eine Lagerung von Proben in Pufferlösung über mehrere Monate ist ebenfalls ohne negative Auswirkungen auf die Strukturerhaltung möglich, jedoch ist für eine längere Lagerung die Einbettung in Kunstharz besser. Für die Kunstharzeinbettung sollten die Muskelfasern in der Orientierung längs und quer ausgerichtet werden. Die daraus angefertigten 0,5–1 μm dicken Kunstharzschnittpräparate (Semidünnschnitte) sind hierbei nicht nur wichtig zur Eingrenzung der relevanten Areale für die Ultradünnschnitte, sondern auch hilfreich, pathologische Veränderungen lichtmikroskopisch zu erkennen [7].

## Muskelerkrankungen im Kindesalter

**Figure 1:**
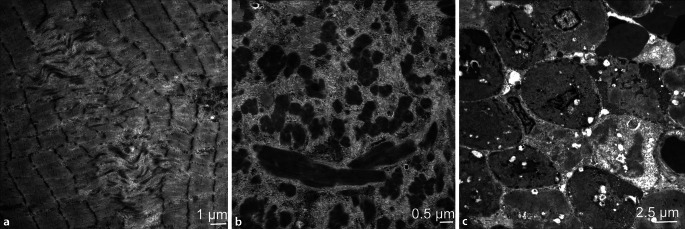

Kongenitale Myopathien (CM) treten meist im frühen Kindesalter auf und sind im Gesamtspektrum der neuromuskulären Krankheiten selten. Die Erkennung, Bezeichnung, nosographische Abgrenzung und muskelbioptische Diagnostik geht auf die Einführung enzymhistochemischer Methoden und der Elektronenmikroskopie in der Myopathologie am Anfang der zweiten Hälfte des vorigen Jahrhunderts zurück [6, 27]. Es werden derzeit 3 Formen unterschieden:

1. Klassische CM, z. B. „core disease“ (CD), Nemalinmyopathie (NM) oder zentronukleäre Myopathie (CNM) wobei jeweils unterschiedliche Gendefekte vorliegen können. Bei der CD stehen ultrastrukturell zentrale, exzentrische oder multiple mitochondrienfreie Läsionen in den Sarkomeren im Vordergrund, welche auch in oxidativen enzymhistochemischen Färbungen erkennbar sind (Abb. 1a). Die NM ist durch die Bildung von Stäbchen („rods“) bzw. Nemalinkörpern in den Muskelfasern gekennzeichnet. „Rods“ sind auch in Semidünnschnitten erkennbar, lassen sich aber nur elektronenoptisch sicher von anderen Einschlüssen, wie z. B. „reducing bodies“ oder „hyaline bodies“, abgrenzen (Abb. 1b; [14]). Eine CNM hat neben zentralisierten Kernen diagnostisch wichtige elektronenmikroskopische Eigenheiten wie „radial strands“ oder „necklace fibers“, welche bei molekular nicht sicher zu interpretierbaren Befunden eine Diagnose erlauben oder bestätigen können (Abb. 1c; [21]).
2. Seltene CM mit bekannten genetischen Befunden wie „reducing body myopathy“, „tubular aggregate myopathy“, „hyaline body myopathy“.
3. Seltene CM ohne bisher bekannten molekularen Hintergrund, wie „cylindral spirals myopathy“ oder „hexagonal crystalline inclusion myopathy“. Eine zusätzliche Gruppe von CM hat sich besonders in den letzten Jahren herauskristallisiert. Diese haben zwar eine bekannte molekulare Genese, aber keine myopathologischen oder gar elektronenmikroskopisch charakteristische Merkmale [20]. Möglicherweise konnten aufgrund der Seltenheit dieser Erkrankungen spezifische myopathologische und elektronenmikroskopische Befunde noch nicht sicher identifiziert werden.

Andere hereditäre neuromuskuläre Krankheiten im Kindesalter, wie z. B. metabolische Erkrankungen oder Muskeldystrophien, zeigen zwar deutliche ultrastrukturelle Veränderungen, weisen aber selten pathognomonische elektronenoptische Befunde auf. Die Diagnose erfolgt daher meist genetisch oder an enzymatischen und immunhistochemischen Reaktionen an Kryostatschnitten.

## Muskelerkrankungen im Erwachsenenalter

**Figure 2:**
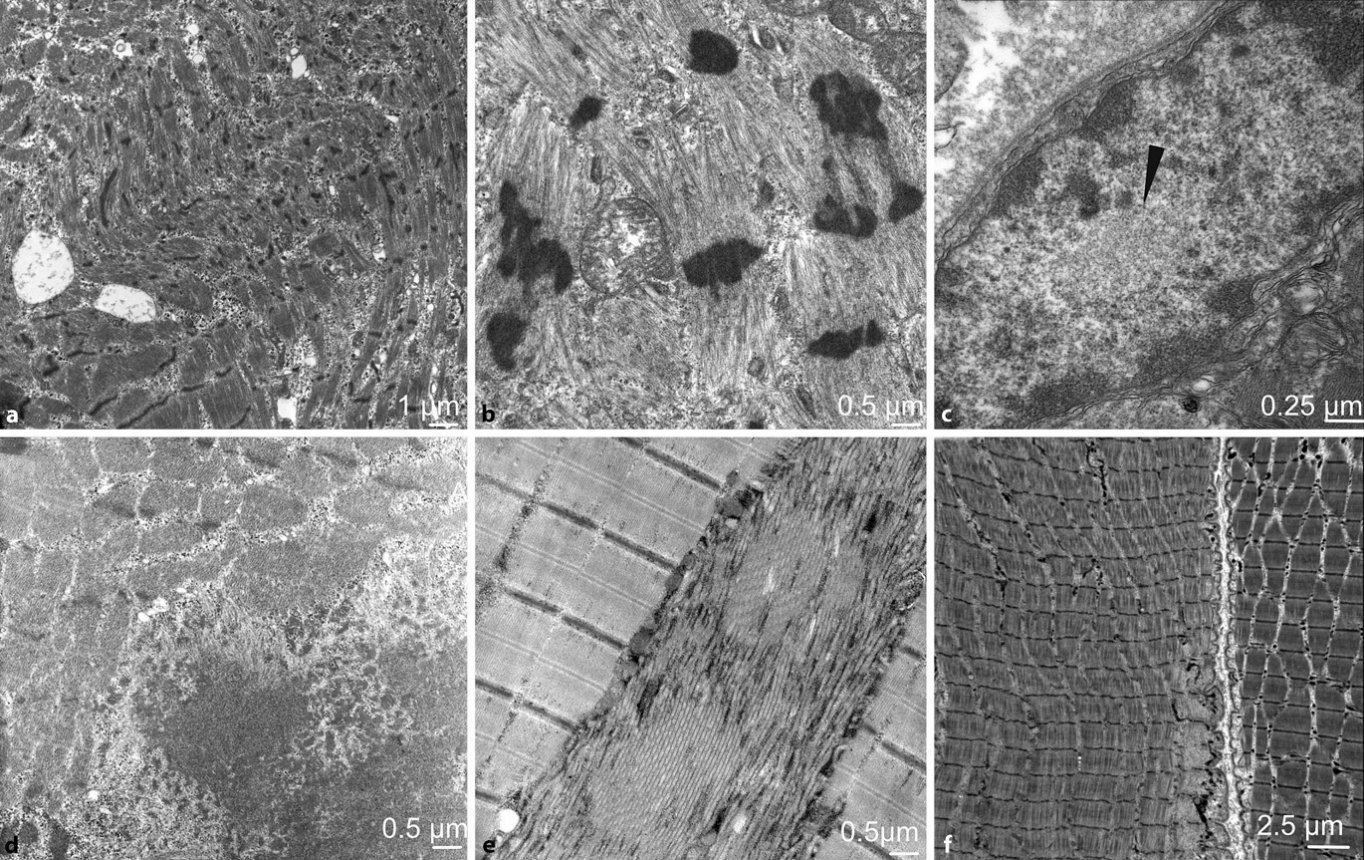

Manche molekularen Varianten einer kongenitalen Myopathie können auch erst im Erwachsenenalter manifest werden und z. B. mit einer Rhabdomyolyse oder malignen Hyperthermie einhergehen [12]. Die ultrastrukturellen Befunde unterscheiden sich dabei nicht von denen kindlicher Erkrankungen (Abb. 2a). Andere Strukturen, wie z. B. „rods“, können dagegen auch bei erworbenen Myopathien auftreten. Eine „sporadic late onset nemaline myopathy“ (SLONM) steht zwar der hereditären NM elektronenmikroskopisch nahe, ist aber immunologischer Natur. Der ultrastrukturelle Nachweis von „rods“ ist hierbei diagnostisch entscheidend und wichtig, da therapeutische Möglichkeiten für diese Erkrankung zur Verfügung stehen (Abb. 2b; [4, 25]).

**Figure 3:**
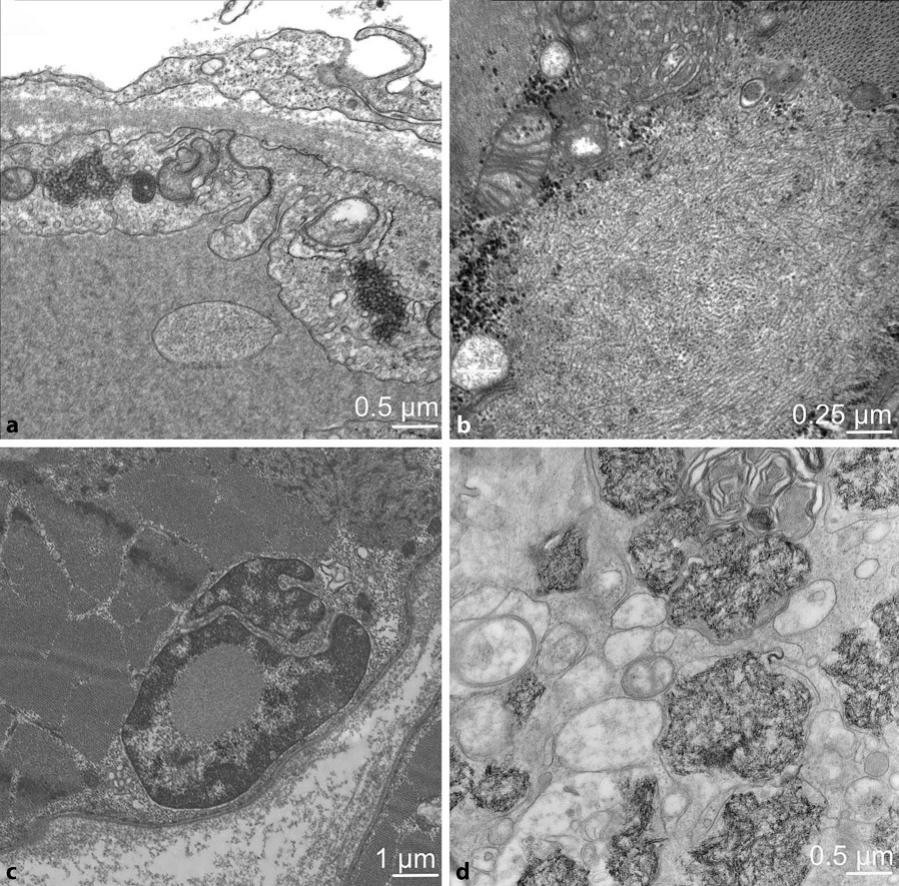

Bei anderen hereditären neuromuskulären Krankheiten im Erwachsenenalter finden sich ultrastrukturell charakteristische Befunde eher selten. Intranukleäre Aggregate von Filamenten sind bei der Diagnose einer okulopharyngealen Muskeldystrophie (OPMD) hilfreich (Abb. 2c). Bei myofibrillären Myopathien (MFM) kommt es durch Mutationen in den Z‑Band-Proteinen Desmin, Alpha-B-Crystallin, ZASP, Myotilin, Filamin‑C und BAG3 zu Aggregaten aus z. B. granulofilamentärem Material, wobei die ultrastrukturelle Einordnung auf die kausale Genmutation hinweisen kann (Abb. 2d; [5, 26]). Myopathien mit tubulären Aggregaten (TA) sind gezeichnet durch morphologisch dicht gepackten Tubuli, die charakteristische Reaktionen in den enzymatischen und immunhistochemischen Präparationen aufweisen und meist subsarkolemmal lokalisiert sind. Die TA sind allerdings nicht pathognomonisch für diese Form der Myopathie, sondern können auch bei anderen hereditären oder erworbenen Erkrankungen auftreten (Abb. 2e; [3]).

Die akute quadriplegische Myopathie aus dem Spektrum der Critical-Illness-Myopathien (CIM) ist durch den selektiven Verlust der dicken Myosinfilamente in den Sarkomeren gekennzeichnet, die am zuverlässigsten elektronenmikroskopisch erfasst werden (Abb. 2f). Obwohl das Elektronenmikroskop gut geeignet ist, motorische Endplatten und deren Morphologie bei z. B. einer Myasthenie oder kongenitalen myasthenischen Syndromen zu beschreiben, sind diese allerdings in proximalen Extremitätenmuskel kaum zu finden.

## Myositis

Die Myositiden sind eine heterogene Gruppe von Erkrankungen, welche sich nach der integrativen Klassifikation in Dermatomyositis (DM), Einschlusskörperchenmyositis (IBM), Anti-Synthetase-Syndrom (ASyS) und immunvermittelte nekrotisierende Myopathie (IMNM) subklassifizieren lassen [1]. Während im höheren Erwachsenenalter die IBM am häufigsten auftritt, gibt es diese Form bei Kindern nicht. Bei Kindern ist die juvenile Dermatomyositis (jDM) der häufigste Subtyp, während die IMNM eine Rarität ist [22]. Es wird hier lediglich auf die ultrastrukturell relevanten Veränderungen eingegangen, welche in der myopathologischen Diagnostik der Myositis hilfreich und mit standardisierten Protokollen und angemessenem Aufwand durchführbar sind.

Für die Diagnose der DM existieren wichtige lichtmikroskopische und immunhistochemische Charakteristika, die in sämtlichen beschriebenen Unterformen erkennbar und in wechselnder Ausprägung zu finden sind [30]. Ultrastrukturell weisen tubuloretikuläre Inklusionen (TRIs, auch undulierende Tubuli genannt) in den Endothelzellen auf eine Schädigung der Kapillaren hin, welche bei nahezu allen Muskelbiopsaten bei einer DM nachweisbar sind und dadurch die Diagnose unterstützen (Abb. 3a). Bei den juvenilen Formen der Myositis lassen sich ebenfalls TRIs in den Endothelzellen, insbesondere bei der jDM nachweisen [22]. TRIs sind allerdings nicht spezifisch für eine DM, sondern können auch bei einer HIV-assoziierten Myopathie oder Overlap-Myopathie bei Lupus erythematodes (LE) auftreten sowie in extramuskulären Organen (z. B. Niere) bei anderen Interferon-assoziierten Erkrankungen.

Für die IBM war lange Zeit der Nachweis ultrastruktureller Veränderungen eine notwendige Voraussetzung für die Diagnosesicherung. Elektronenmikroskopie ist in der aktuellen Klassifikation keine Voraussetzung, um eine „sichere“ IBM zu diagnostizieren, hilft aber, die Diagnose zu sichern. In der IBM sind sog. „rimmed vacuoles“ in Randarealen der Muskelfasern als basophile Vakuolen in den HE-gefärbten Muskelfasern erkennbar, welche sich ultrastrukturell als autophagische Vakuolen mit myelinähnlichen Strukturen (MLB) darstellen. Diese Strukturen sind allerdings unspezifisch und können auch in denervierten Muskelfasern und bei distalen Myopathien auftreten. Darüber hinaus finden sich bei der IBM Tubulofilamente häufiger im Zytoplasma oder in der Nähe von Vakuolen, die oft mit verwirbeltem Zelldetritus einhergehen. Diese Filamente bestehen aus 15–17 nm (150–170 A) paarigen, helikal verwirbelten Filamenten, welche an Tau-Protein erinnern [10]. Diese Filamente können auch im Zellkern auftreten und sind manchmal von fragmentiertem Euchromatin schwer abzugrenzen (Abb. 3b). Diese Kerneinschlüsse sollten auch nicht mit anderen intranukleären Einschlüssen verwechselt werden, welche für das ASyS pathognomonisch sind (Abb. 3c; [29]).

Für die IMNM sind bislang keine pathognomonischen ultrastrukturellen Veränderungen beschrieben.

Bei rheumatischen Erkrankungen kann es zu einer Mitbeteiligung der Skelettmuskulatur kommen. Bei der systemischen Sklerose wiesen 2 unabhängige Studien auf ultrastrukturelle Veränderungen der Kapillaren, welche vor allem die Basalmembranen und die Perizyten betreffen, hin [15, 28]. Ob diese Veränderungen in der Zukunft diagnostische Bedeutung haben, muss in weiteren Studien validiert werden.

Bei der durch Impfungen mit aluminiumhaltigen Impfstoffen induzierten Makrophagen-Myofasziitis (MMF) zeigen die Makrophagen der myofaszialen Grenzzone ultrastrukturell charakteristisches, reichlich feingranuläres Aluminium in Form von Spiculi, die innerhalb der lysosomalen Kompartimente erkennbar sind (Abb. 3d). Histologisch sind diese als diastaseresistente Ablagerungen in der PAS-Färbung darstellbar und sind in der Saure-Phosphatase-Reaktion kräftig positiv [9].

## Mitochondriale Veränderungen

**Figure 4:**
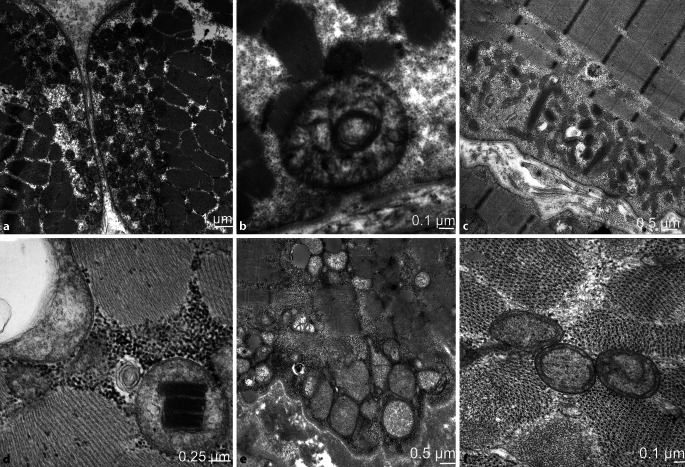

In der Skelettmuskulatur können die Mitochondrien bei hereditären mitochondrialen Erkrankungen, aber auch bei anderen genetischen und erworbenen Muskelerkrankungen pathologisch verändert sein. Daher müssen mitochondriale Veränderungen immer im Kontext mit den klinischen und genetischen Befunden und der Muskelhistologie beurteilt werden. Bei einem Verdacht auf eine genetische mitochondriale Erkrankung kann die ultrastrukturelle Untersuchung hilfreich sein, da histomorphologisch nicht immer pathologische Befunde wie COX-negative Muskelfasern oder mitochondriale Aggregate in den enzymatischen Präparationen auftreten müssen. Bei Dysfunktion von Mitochondrien kann es zu einer Vermehrung und Aggregatbildung kommen sowie zu einer Polymorphie sowie Störung und Auflösung der Cristae (Abb. 4a–e). Parakristalline Einschlüsse können sowohl bei hereditären, aber auch erworbenen Erkrankungen auftreten und sind daher nicht als pathognomonisch zu werten (Abb. 4c,d).

## Vakuoläre Strukturen

Die quergestreifte Muskulatur ist starken Zugkräften ausgesetzt und es fallen Proteinabbauprodukte an, welche mithilfe von autophagischen Prozessen entsorgt und recycelt werden [2]. Die Detektion einer gesteigerten oder gestörten Autophagie in der Muskelbiopsie ist unspezifisch und wird in unterschiedlichen Muskelerkrankungen beschrieben [17]. In den HE-Kryoschnitten stellen sich autophagische Vakuolen häufig mit basophilem granulärem Material dar. Hilfreich sind eine immunhistochemische Färbung mit den Autophagiemarkern p62 und LC3 sowie PPD-gefärbte Semidünnschnitte von in Kunstharz eingebettetem Gewebe, die größere Autophagosomen als dunkel angefärbte Vakuolen darstellen. Ultrastrukturell sind Autophagosomen von einer Doppelmembran umgeben und enthalten elektronendichtes Material oder Organellen, wobei durch Fixationsartefakte die Doppelmembran nicht immer sicher zu erkennen ist. Andere vakuoläre Strukturen wie aufgeblähte Mitochondrien können fälschlicherweise als Autophagosomen gedeutet werden [8].

**Figure 5:**
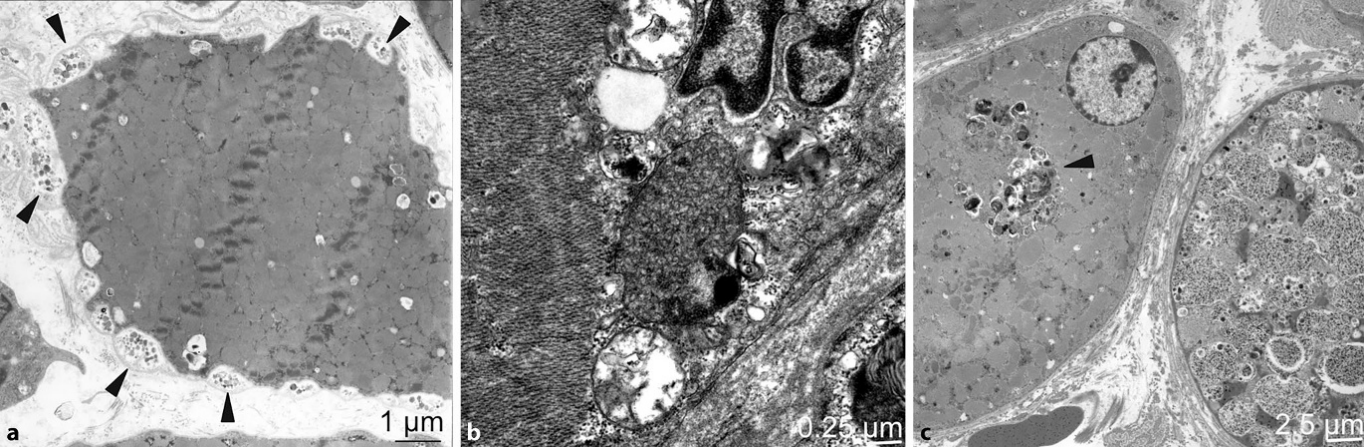

Die Analyse von Vakuolen kann die Pathologie genetischer und erworbener Myopathien weiter einzugrenzen, wobei die Vakuolen ultrastrukturell besser beurteilbar sind [16]. Bei den erworbenen Myopathien unterstützt der Nachweis von „rimmed vacuoles“ die Diagnose einer IBM (Abb. 5a). Vakuolen können auch durch toxische Substanzen verursacht sein. Hierbei spielt insbesondere die Therapie mit Chloroquin/Hydroxychloroquin eine Rolle, welche als immunmodulierender Wirkstoff bei rheumatischen Erkrankungen eingesetzt wird [19]. Bei der Chloroquin-Myopathie finden sich Vakuolen mit charakteristischen ultrastrukturellen kurvilinearen Profilen, durch welche die Diagnose gesichert werden kann (Abb. 5b).

Unterschiedliche hereditäre Myopathien können mit ausgeprägten autophagischen Vakuolen einhergehen, wie z. B. *LAMP2* („Danon disease“), *EPG‑5* (Vici-Syndrom) oder *VMA21* („X-linked myopathy with excessive autophagy“).

Morbus Pompe ist eine Glykogenspeichererkrankung (GSD II), welche durch einen Mangel des lysosomalen Enzyms α‑1,4‑Glucosidase verursacht wird und im Säuglings- und Erwachsenenalter auftreten kann. Diagnostisch spielt die Muskelbiopsie keine große Rolle mehr und wurde von Trockenbluttest und Next Generation Sequencing (NGS) abgelöst. Neben lysosomalen und extralysosmalen Glykogenablagerungen zeigt sich eine gesteigerte Autophagie mit teils großen Vakuolen, wobei die Muskelfasern sehr unterschiedlich stark betroffen sein können (Abb. 5c; [13]).

## Kardioskelettaler Link bei muskulären Erkrankungen

Skelett- und Herzmuskelgewebe haben gemeinsame Merkmale, weshalb Mutationen vieler Gene, die für Skelettmuskelerkrankungen verantwortlich gemacht werden, auch kardiale Symptome verursachen. Es kann dabei entweder die Beteiligung der Skelettmuskulatur oder der Herzmuskultur im Vordergrund stehen, wobei insbesondere im Kindesalter eine hereditäre Muskelerkrankung zu einer ausgeprägten Kardiomyopathie mit nachfolgender Herzinsuffizienz führen kann. Die pathologische Diagnostik am Herzmuskel wird in der Regel an formalinfixiertem Gewebe durchgeführt. Zur erweiterten muskulären Diagnostik hat sich eine Aufarbeitung an nativem Gewebe entsprechend einer Skelettmuskelbiopsie einschließlich einer ultrastrukturellen Analyse bewährt [23].

**Figure 6:**
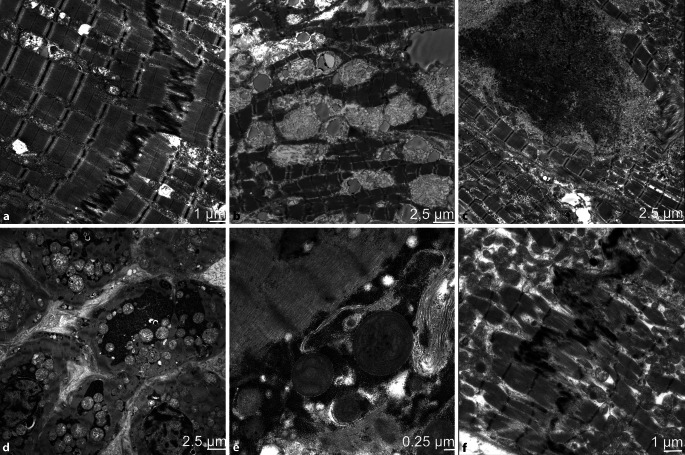

Für eine morphologische Beurteilung des Herzmuskels sind insbesondere die Sarkomerstruktur, Mitochondrien, Glanzstreifen/„intercalated disks“ (ID), Zellkerne und Speicherprodukte wichtig (Abb. 6a). Eine Störung der Sarkomerenstruktur gibt Hinweise auf einen Funktionsverlust der Herzmuskelfaser und kann sich darstellen als Auflösung, Z‑Band-Veränderung und unterschiedliche Dicke und Länge der Sarkomere [18]. Bei vielen metabolischen Erkrankungen zeigt sich eine kardiale Beteiligung und es lassen sich spezifische Speicherprodukte in den Kardiomyozyten nachweisen (Abb. 6b). Pathologische Speicherprodukte sollten insbesondere bei ungeklärtem plötzlichem Kindstod im Herzmuskel untersucht werden, da diese in Formalin fixierten Proben mit konventionellen Färbemethoden übersehen werden können. Sarkomerenmaterialähnliche Aggregate und eine gestörte Autophagie mit Autophagosomen können, z. B. bei Proteinaggregatmyopathien, auftreten (Abb. 6c; [23, 24]). Die Zellkerne stellen sich bei Kardiomyopathien häufig polymorph und entrundet dar.

Mitochondrien treten beim Herzmuskel im Vergleich zum Skelettmuskel in größerer Anzahl auf. Sekundäre unspezifische Veränderungen wie eine Vermehrung mit Aggregatbildung (meist perinukleär) und eine Polymorphie sind von einer solchen genetischen mitochondrialen Erkrankung häufig nicht sicher abzugrenzen. Eine deutliche Auflösung der Cristae mit Darstellung von parakristallinen Einschlüssen und eine deutliche Polymorphie können allerdings eine hereditäre Erkrankung bestätigen (Abb. 6d,e). Eine genetische Abklärung sollte in jedem Fall erfolgen, um die Verdachtsdiagnose zu sichern. Hierbei kann auch die Analyse von unfixiertem Herzmuskelgewebe hilfreich sein. Die IDs sind hochkomplexe Strukturen, welche eine wichtige Funktion zur Kommunikation der einzelnen Zellen haben, wodurch das Herzgewebe als funktionelles Synzytium wirken kann. Die IDs können bei Kardiomyopathien eine Irregularität aufweisen ([11]; Abb. 6f).

Zusammenfassend kann die ultrastrukturelle Analyse des Herzmuskels das Ausmaß der pathologischen Veränderungen gut dokumentieren und die Diagnose einer Kardiomyopathie unterstützen. Insbesondere bei hereditären Kardiomyopathien kann der Nachweis spezifischer Veränderungen hilfreich sein, die zugrunde liegende Pathogenese besser zu erfassen [23, 24].

**Table 1:** 

	Unterstützend	Beweisend	Unspezifisch
*Muskelerkrankungen im Kindesalter*			
Kongenitale Myopathien		„cores“, „rods“	–
Muskeldystrophie	–	–	Keine spezifischen Befunde
*Muskelerkrankungen im Erwachsenenalter*			
Kongenitale Myopathien mit später Manifestation		„cores“, „rods“	–
„Sporadic late onset nemaline myopathy“ (SLONM)	–	rods	–
Okulopharyngeale Muskeldystrophie (OPMD)	–	Nukleäre filamentöse Aggregate	–
Myofibrilläre Myopathie (MFM)	–	Granulofilomentöse Aggregate	–
Myopathien mit tubulären Aggregaten	Tubuläre Aggregate	–	–
Critical-Illness-Myopathie (CIM)	–	Verlust der dicken Myosinfilamente	–
*Myositis*			
Dermatomyositis (DM)	Tubuloretikuläre Inklusionen	–	–
Einschlusskörperchenmyositis (IBM)	„Rimmed vacuoles“, Tubulofilamente, pathologische Mitochondrien	–	–
Anti-Synthetase-Syndrom (ASyS)	Tubuloretikuläre Inklusionen	Intranukleäre Einschlüsse	–
Immunvermittelte nekrotisierende Myopathie (IMNM)	–	–	Keine spezifischen Befunde
Systemische Sklerose	Basallaminaverdickung	–	–
Makrophagen-Myofasziitis (MMF)	–	Feingranuläre lysosomale Einschlüsse	–
*Genetische Mitochondriopathien*	Pathologische Mitochondrien	–	–
*Vakuoläre Myopathien*	Autophagische Vakuolen	–	–
*Kindliche Kardiomyopathien*	Pathologische Mitochondrien, Störung der Sarkomere und Glanzstreifen, Autophagische Vakuolen	Granulofilomentöse Aggregate	–

## Fazit für die Praxis

- Bei der Fragestellung einer Muskelerkrankung sollte bei einer Muskelbiopsie immer Gewebe für die elektronenmikroskopische Analyse bereitgestellt werden.
- Die ultrastrukturelle Begutachtung ist hilfreich, eine Diagnose zu stützen oder die Pathogenese besser einzugrenzen und weist bei einigen Muskelerkrankungen pathognomonische Veränderungen auf.
